# Hemoglobin Disorders in South India

**DOI:** 10.5402/2011/748939

**Published:** 2011-06-28

**Authors:** Vani Chandrashekar, Mamta Soni

**Affiliations:** Department of Hematology, Apollo Hospitals, 21, Greams Lane, Off Greams Road, Chennai 600006, India

## Abstract

Cation exchange-high performance liquid chromatography (CE-HPLC) is increasingly being used as a first line of investigation for hemoglobinopathies and thalassemias. Together with a complete blood count, the CE-HPLC is effective in categorizing hemoglobinopathies as traits, homozygous disorders and compound heterozygous disorders. We carried out a one year study in Apollo Hospitals, Chennai (Tamil Nadu, South India) during which 543 abnormal chromatogram patterns were seen. The commonest disorder we encountered was **β**-thalassemia trait (37.9%), followed by HbE trait (23.2%), homozygous HbE disease (18.9%), HbS trait (5.3%), HbE **β**-thalassemia (4.6%), HbS **β**-thalassemia (2.5%), **β**-thalassemia major (2.3%), HbH (1.6%), homozygous HbS (1.4%), HbD trait (0.7%). The average value of HbA2 in **β**-thalassemia minor was 5.4%. **β**-thalassemia major had an average HbF of 88% and in HbH the mean A2 was 1.4%. Among the HbE disorders the HbA2 + HbE was 30.1% in the heterozygous state, 90.8% in the homozygous state and 54.8% in HbE **β**-thalassemia. In the sickle cell disorders, HbS varied from 30.9% in the trait to 79.9% in the homozygous state to 65.6% in HbS **β**-thalassemia.

## 1. Introduction

Hemoglobinopathies result from a structural defect in the globin gene, whereas the thalassemias are due to a quantitative defect in the globin chain production. The average frequency of HbS and HbD is 4.3% and 0.86%, respectively in Indian population [1]. HbS, HbE, and HbD are prevalent in India [1]. HbE gene frequency in north eastern regions of India has been reported to be 10.9% [1]. In a study, the prevalence of sickle cell disorders was found to vary from 2.4% to 5.6% among the tribes of Orissa in eastern India [2]. In central India, the prevalence of sickle cell disorders was observed to be 5.7% among children [3]. The frequency of HbD is around 0.5–3.1% in Uttar Pradesh [4].

Hemoglobin separates into major and minor hemoglobins when subjected to CE-HPLC. The order of elution of the various components is HbA1a, HbA1b, HbF, LA1c/CHb-1, LA1c/CHb-2, HbA1c, P3 (Hbd component), HbA0, and HbA2. The minor hemoglobins A1a, A1b, A1c, F1, and the P3 component are posttranslational modifications of the globin chains. HbA2, a minor hemoglobin, however, is composed of two alpha and two delta chains. HbA0 and HbF are the major hemoglobins in a normal hemolysate. 

 An elevated HbA2 with an average value of about 5%, along with microcytic hypochromic indices, is characteristic of *β*-thalassemia trait [5]. In *β*-thalassemia major, in addition to a markedly microcytic hypochromic blood picture, there are elevated HbA2 and elevated HbF ranging from 10 to 90% [5]. HbE trait is diagnosed by the presence of a high HbA2 (E+A2), approximately 30% [6]. Homozygous HbE patients have approximately 90% HbE+A2 with minor elevation of HbF [6]. HbE+A2 levels of 40–60% with marked elevation of HbF are seen in HbE-*β*-thalassemias [6]. HbS is around 40% in sickle cell trait, 90–95% in sickle cell anemias (which varies inversely with HbF proportion), and less than 50% in sickle *β*-thalassemias [7]. Approximately less than 50% of abnormal hemoglobin is seen in HbD traits [7].

The present study highlights the detection of the hemoglobinopathies and thalassemias by CE-HPLC. We undertook this study as there is limited available data on large scale studies of hemoglobinopathies in South India. Large studies on the spectrum of hemoglobinopathies on the Bio-Rad D-10 analyser have not been reported previously. This study is a one-year study, carried out in Apollo Hospitals, Chennai, a tertiary care hospital in South India, and includes all patients who had a clinical or familial suspicion of hemoglobinopathy and a hemoglobinopathy work up was ordered for diagnostic purposes.

## 2. Materials and Methods

All patients for whom a CE-HPLC was requested during the period from December 2009 to November 2010 were included in this study. Patients with a recent history of transfusion (three months prior to sample collection) were excluded from the study. A complete blood count (LH-750 analyzer) for red cell indices, a peripheral smear for red blood cell morphology, and an HbH preparation for HbH inclusions were studied for all the patients. HbH smear was prepared by incubating equal volume of blood with new methylene blue for two hours at 37°C and then preparing a smear similar to that for reticulocyte count. HbH inclusions (“golf ball” inclusions) are seen as numerous, large pale blue inclusions distributed evenly in the red cells. A sickling test using the reducing agent sodium metabisulfite was prepared for cases with an abnormal hemoglobin in the S window of the chromatogram. The blood samples collected in EDTA vacutainer were diluted and injected in to the analytical cartridge of D-10 analyzer (Bio-Rad Laboratories, Hercules, CA). Phosphate buffers of increasing strength are then pumped in to the cartridge and the hemoglobins elute out based on their ionic interactions with the cartridge. The elute flows through the flow cell of the filter photometer where their absorbance at 415 nm is recorded. A chromatogram for each sample is obtained using the HbA2/HbF/HbA1c dual program. A recorder pack containing the elution buffers, calibrators, calibrator diluents, whole blood primer, and sample vials are provided with each kit. The hemoglobins fall into windows which are defined by their retention times. Hemoglobins with retention times outside the windows are detected as unknown peaks. The reference ranges for HbA2 and HbF in our laboratory for adults are 1.8–4% and less than 1, respectively. 

## 3. Observations

**Figure 1:**
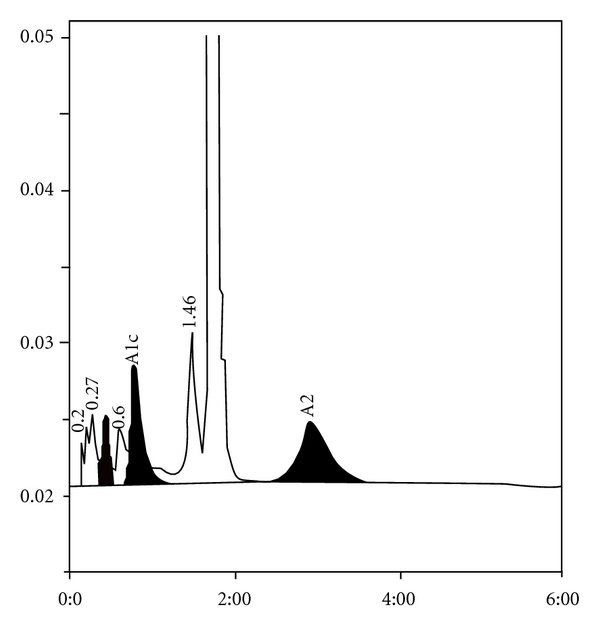
*β*-thalassemia trait with HbA2 of 5.6 and HbF of 1.2%.

**Figure 2:**
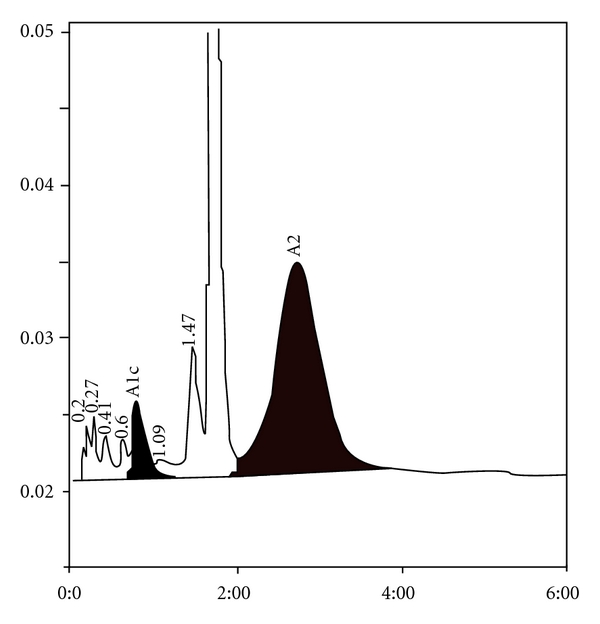
HbE trait with HbA2 and HbE in the same window and HbE+A2 amounting to 31.7%.

**Figure 3:**
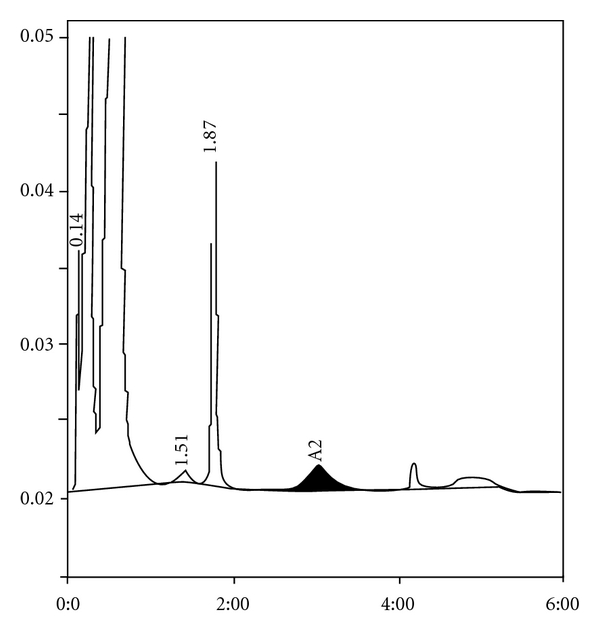
*β*-thalassemia major with elevated HbF.

**Figure 4:**
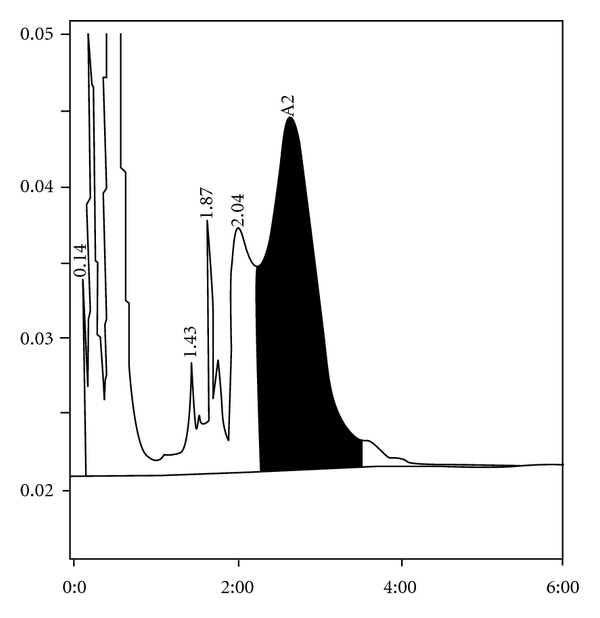
HbE *β*-thalassemia with elevated HbF falling in the LA1c/CHb-1 peak. HbE+A2 was 41.6% and HbF 35.7%.

**Figure 5:**
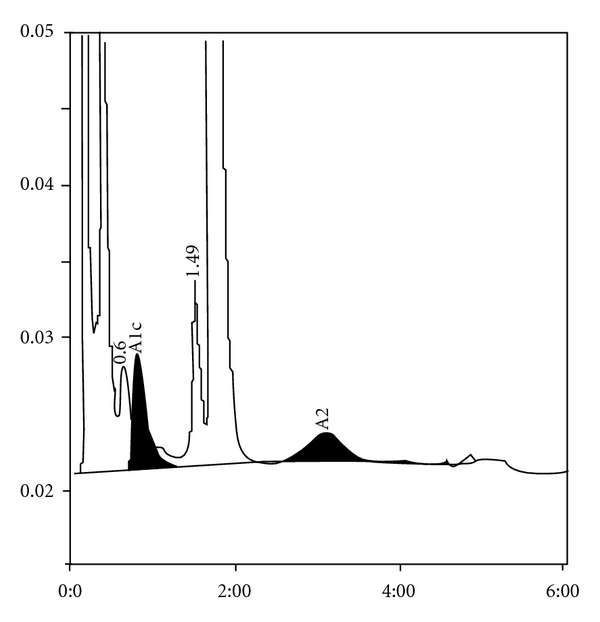
HbH with HbA2 of 1.1%, elevated HbA1a, and an unknown peak at 0.38 minutes.

**Figure 6:**
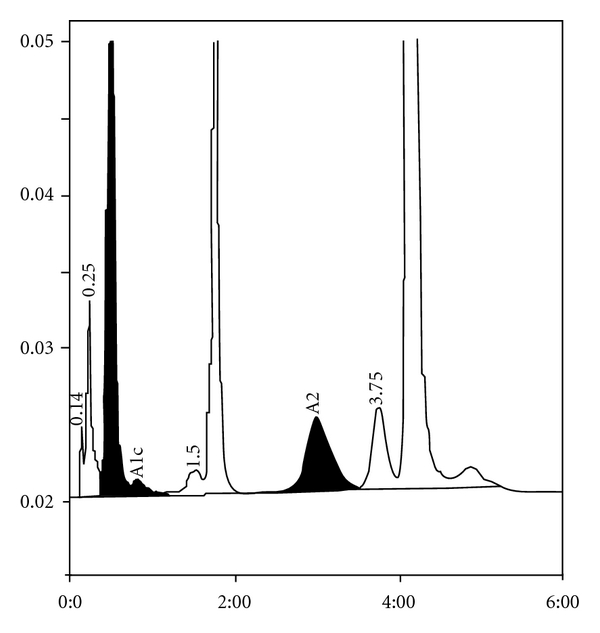
HbS *β*-thalassemia with elevation of HbF and HbA2.

There were five hundred and forty three abnormal chromatograms (see Figures 1, 2, 3, 4, 5, and 6). Of these, 206 (37.9%) were *β*-thalassemia trait, 126 (23.2%) HbE trait, 103 (18.9%) homozygous HbE, 29 (5.3%) HbS trait, 25 (4.6%) HbE *β*-thalassemia, 14 (2.5%) HbS *β*-thalassemia, 13 (2.3%) *β*-thalassemia major, nine (1.6%) HbH, eight (1.4%) homozygous HbS, four (0.7%) HbD trait, one (0.1%) each of homozygous HbD disorder, HbJ trait, HbLepore trait, hereditary persistence of fetal hemoglobin, and HbSC and HbSE disorder. Of these, 289 (53.2%) were males and (46.7%) 254 females.  Table 1 shows the distribution of the common hemoglobin disorders among children and adults in our study. Among our patients, 16.5% were less than 12 years and in both groups *β*-thalassemia trait was the commonest abnormality.

### 3.1. Heterozygous Hemoglobinopathies (E, S, D Traits)

The lowest average hemoglobin was seen in HbD trait. The lowest average MCV, MCH, and MCHC was seen in HbE trait. The highest percentage of abnormal hemoglobin was seen in HbD trait (Table 2). The single case of J trait had a P3 peak of 22.5% (HbJ presents as an elevated P3 peak).

### 3.2. Compound Heterozygous Disorders (E *β*-Thalassemia and S *β*-Thalassemia)

The lowest average hemoglobin, MCV, MCH, and MCHC were seen in HbE Beta-thalassemia. The average abnormal hemoglobin was more in HbS *β*-thalassemia compared to HbE *β*-thalassemias (65.6 versus 54.8), whereas it was contrary to the amount of HbF (19.2 versus 31.3) (Table 3).

### 3.3. Homozygous Disorders (SS, EE)

A lower average hemoglobin was seen in HbSS, whereas the percentage of abnormal hemoglobin was higher in HbEE. The average HbF was, however, higher in HbSS (Table 4).

### 3.4. HbE Disorders (E Trait, EE, E *β*-Thalassemia)

Comparing all the HbE disorders, it is seen that the lowest hemoglobin as well as the highest proportion of HbF is seen in HbE *β*-thalassemia (Table 5). The single case of HbSE disorder had a hemoglobin of 10.1 gm%, thalassemic indices with MCV of 71fl, MCH of 23.1 pg, 36.7% of HbA2+HbE, and 2.8% of HbF.

### 3.5. Sickle Cell Disorders (S Trait, SS, S *β*-Thalassemia)

Unlike the HbE disorders, homozygous HbS disorder had the lowest hemoglobin (Table 6). The single case of HbSC disorder had a hemoglobin of 13.8 gm% and 47.1% HbS, whereas HbS was 53.4% in HbSE disorder. The patients' HbC eluted in the C window at 4.79 minutes and constituted 45.8% of the total hemoglobin. These values of HbS are higher than seen in HbS trait where the average was 30.9%.

 The highest average HbA2 of 4.2% was seen in HbS *β*-thalassemias (Table 7).

### 3.6. Thalassemias (*β*-Thalassemia Trait, *β*-Thalassemia Major, *α*-Thalassemia)

On comparing the thalassemic disorders in our study (*β*-thalassemia trait, *β*-thalassemia major, and *α*-thalassemias), we observed that the lowest average hemoglobin was seen in *β*-thalassemia major. The lowest average MCV was seen, however, in *β*-thalassemia trait, whereas the lowest average MCH and MCHC was recorded in *α*-thalassemias (Table 8). The average HbF was 88% in *β*-thalassemia major and less than one in *α*-thalassemias (HbH).

 All the cases of *α*-thalassemias included in our study had numerous HbH inclusions. The single case of HbLepore trait had a hemoglobin of 6.6 gm%, MCV of 52.9fl, MCH of 15.8 pg, an elevated HbF of 13.7%, and elevated HbA2 of 10.6%. One case of hereditary persistence of fetal hemoglobin was noted with a hemoglobin of 13.3 gm%, normal red cell indices, and an HbF of 36.2%.

### 3.7. Minor Hemoglobins (Tables 9, 10, and 11)

The highest mean HbA1a was seen in *α*-thalassemias and the lowest was noted in HbD trait. HbE *β*-thalassemia had the highest mean HbA1b and the lowest was seen in homozygous HbE disorder. HbA2 was noted to be highest in *β*-thalassemia trait, and lowest in *α*-thalassemias (excluding the HbE disorders where HbE and HbA2 fall in the same window and A2 is not detected separately).

### 3.8. Retention Times

All our cases of HbH in addition to having a high HbA1a with a characteristic retention time of 0.16 minutes also had an unknown peak eluting at 0.37–0.38 minutes. The average retention time of HbA1a was 0.21 (range from 0.19 to 0.25, except in *α*-thalassemias where it was 0.16 minutes). The average retention time of HbA1b was 0.28 with a range from 0.25 to 0.35, and HbA1c average was 0.79, with a range of 0.71–0.97 minutes. The retention time of HbF was 0.43, ranging from 0.41 to 0.51 in all cases where HbF was normal or minimally elevated. In HbE *β*-thalassemias, the average for HbF was 0.54 minutes, whereas in HbS *β*-thalassemias it was 0.51. The average retention time of P3 peak was 1.45, with a range from 1.41 to 1.51 with the exception of the HbJ trait where it was 1.33 minutes. HbA2 retention time ranged from 2.74 to 3.09 with an average of 2.94. The average retention time of HbA2 in the HbE disorders was 2.76, 2.7, and 2.71 in HbE trait, HbEE disorder, and HbE *β*-thalassemia, respectively. The retention time for HbS ranged from 4.04 to 4.18 in HbS trait with an average of 4.13. In HbSS disorder, the average was 4.08 and it was 4.10 in HbS *β*-thalassemias. HbD had an average retention time of 3.75 with a range from 3.72 to 3.82. In homozygous HbD, the retention time of the abnormal hemoglobin was noted to be 3.77 minutes.

## 4. Discussion

In our study, the commonest disorder was *β*-thalassemia trait (37.9%), followed by HbE trait (23.2%), homozygous HbE (18.9%), HbS trait (5.3%), HbE *β*-thalassemia (4.6%), HbS *β*-thalassemia (2.5%), *β*-thalassemia major (2.3%), HbH (1.6%), homozygous HbS (1.4%), and HbD trait (0.7%). This compares with other studies [8, 9] (Table 12) where *β*-thalassemia trait is the commonest disorder. In the study by Balgir [10], sickle cell trait was noted to be common as the study included tribes from Orissa where this gene is prevalent. Our study had a high proportion of HbE disorders due to the many patients from north eastern regions of India who frequent our hospital for treatment.

 The average value of HbA2 was 5.4% in *β*-thalassemia traits compared to 5% in other studies [5]. HbF was minimally elevated in 42% of our *β*-thalassemia trait patients and was around 1.4%. HbF ranges from 10 to 90% in *β*-thalassemia major [5]. In our study, the HbF average was 88%. HbA2 may be elevated in *β*-thalassemia major and in our study 15% of the patients with *β*-thalassemia major were seen to have elevated HbA2 and the average value of HbA2 was around 3.1%.

 In the Bio-Rad Variant analyser, HbH can only be suspected by the presence of peaks at the beginning of the chromatogram. In the D-10 analyser, we have seen that in addition to an elevation of HbA1a with a characteristic retention time of 0.16 there was an unknown peak at 0.37–0.38 minutes. All our cases of HbH had low HbA2 with an average of 1.4%, and numerous HbH inclusions.

 HbE+A2 has been reported to be around 30% in HbE trait [6]. In our study, the average was 30.1% with a minimal elevation of HbF of 1.32%. HbE+A2 has been reported to be around 90% with minimal elevation of HbF in homozygous HbE, and it is about 40–60% in HbE *β*-thalassemias [6]. In our study, HbE+A2 was 90.8% in homozygous HbE and 54.8% in HbE *β*-thalassemias. HbF was 3.7% in homozygous HbE and 31.3% in HbE *β*-thalassemias. 

HbS has been reported to be around 40% in trait, 90–95% in homozygous HbS, and less than 50% in HbS *β*-thalassemias [7]. In our cases, it was 30.9% in trait, 79.9% in the homozygous forms, and 65.6% in HbS *β*-thalassemia. On comparing HbE *β*-thalassemia and HbS *β*-thalassemia disorders, it is seen that the percentage of abnormal hemoglobin was more in HbS *β*-thalassemia than HbE *β*-thalassemias and the converse was seen for HbF (31.3 in E disorder and 19.2 in S disorder). However, the homozygous disorders of the same showed a higher abnormal hemoglobin in homozygous HbE (90.8%) with a lower average HbF (3.7%) when compared to homozygous HbS where it was 79.9% and 8%, respectively. The lowest average hemoglobin among these four disorders was seen in homozygous HbS (7.8%). The reason for this could be that the sickle cell disorders are hemolytic disorders, whereas the HbE disorders are somewhat similar to thalassemic disorders (due to slower production), and hence in the homozygous form HbS is noted to have the lowest hemoglobin. It is interesting to note that among the HbE disorders HbE *β*-thalassemia had the lowest hemoglobin and also the highest proportion of HbF. This does not reflect the clinical diversity of this disorder where highest HbF is noted in patients with a higher hemoglobin level. In all our cases of HbE disorders, the levels of HbA2 could not be evaluated as the presence of HbE is suspected by the presence of a abnormally high HbA2 peak. HbLepore, HbD-Iran, and others such as HbHonolulu also present as a raised HbA2 peak and the ethnicity along with the amount of abnormal hemoglobin is taken in to account before labeling these patients. HbLepore has been reported to have a characteristic hump in the HbA2 peak. However, in our patient it was not seen and the HbA2 was elevated to 10.6%. The level of HbD has been reported to be less than 50% in traits, and in our cases the average was 35.4%. 

Levels of all the minor hemoglobins have not been reported in previous studies. Maximal elevation of minor hemoglobin HbA1a (7.6) was seen in HbH and of HbA1b (6.5) was seen in HbE *β*-thalassemia. The significance of this is not known. The retention times on the D-10 analyser have not been reported previously and to the best of our knowledge this is the first study detailing the retention times. After a detailed search, we have found only one large-scale study on the D-10 analyser [11], and to the best of our knowledge this is the first report from South India.

 In addition to HbA2 being unrecordable in HbE disorders, the other drawback is the inability to measure HbA1c in presence of high HbF levels (15% and more). The elevated HbF then presents as an elevated HbA1c or elevated LA1c (the labile portion of A1c) and other methods have be to used to evaluate the same. 

## Figures and Tables

**Table 1:** Distribution of hemoglobin disorders in adults and children.

Disorder	12 years and less (%)	More than 12 years (%)
*β*-thalassemia trait	4.7	35
HbE trait	3.6	19.5
Homozygous HbE	2.3	16.5
HbS trait	0.7	4.6
HbE *β*-thalassemia	1.8	2.7
HbS *β*-thalassemia	0.7	1.8
*β*-thalassemia major	2.3	—

**Table 2:** Heterozygous hemoglobinopathies.

Disorder	No. of cases	Hb gm%	MCV (fl)	MCH (pg)	MCHC (g/dL)	RDW%	RBC × 10^6^/cumm	Abnor Hb%
E trait	126	10.3	75.5	24.2	31.7	16.1	4.3	30.1
S trait	29	11.3	77.2	25.1	32.3	15.6	4.5	30.9
D trait	4	10	82.6	27.3	33	17.2	3.6	35.4

**Table 3:** Compound heterozygous disorders.

Disorder	No. of cases	Hb (gm%)	MCV (fl)	MCH (pg)	MCHC (gm/dL)	RDW (%)	RBC × 10^6^ cumm	%Abnor Hb	HbF (%)
E *β*-thalassemia	25	6.9	67.2	19.7	29.2	26.6	3.6	54.8	31.3
S *β*-thalassemia	14	9	73.8	22.8	30.7	18.9	4.1	65.6	19.2

**Table 4:** Homozygous hemoglobinopathies.

Disorder	No. of cases	Hb (gm%)	MCV (fl)	MCH (pg)	MCHC (gm/dL)	RDW (%)	RBC × 10^6^ cumm	Abnormal Hb	%HbF
HbEE	103	9.8	63.6	20.4	31.9	18	4.8	90.8	3.7
HbSS	8	7.8	77.4	24.5	31.5	24.8	3.3	79.9	8

**Table 5:** HbE disorders.

Disorder	No. of cases	Hb (gm%)	MCV (fL)	MCH (pg)	MCHC (gm/dL)	RDW (%)	RBC × 10^6^ cumm	Abnormal Hb %	%HbF
HbE trait	126	10.3	75.5	24.2	31.7	16.1	4.3	30.1	1.32
HbEE	103	9.8	63.6	20.4	31.9	18	4.8	90.8	3.7
HbE *β*-thalassemia	25	6.9	67.2	19.7	29.2	26.6	3.6	54.8	31.3

**Table 6:** HbS disorders.

Disoder	No. of cases	Hb (gm%)	MCV (fl)	MCH (pg)	MCHC (gm/dL)	RDW (%)	RBC × 10^6^ cumm	Abnormal Hb %	%HbF
HbS trait	29	11.3	77.2	25.1	32.3	15.6	4.5	30.9	1.34
HbSS	8	7.8	77.4	24.5	31.5	24.8	3.3	79.9	8
HbS *β*-thalassemia	14	9	73.8	22.8	30.7	18.9	4.1	65.6	19.2

**Table 7:** HbA2 in sickle cell disorders.

Disorder	A2 levels (%)
HbS trait	3.3
HbSS	2.7
HbS *β*-thalassemia	4.2

**Table 8:** Comparison of thalassemias (—indicates not detected).

Disorder	No. of cases	Hb (gm%)	MCV (fl)	MCH (pg)	MCHC (gm/dL)	RDW (%)	RBC × 10^6^ cumm	Hb F%	Hb A%
*β* thal trait	206	10.4	65.5	19.9	30.5	16.5	5.2	1.4	81.3
*β* thal major	13	4.8	67.9	21.1	31	30.9	2.4	88	7.2
*α* thal	9	8.3	67.2	18.4	28.2	23.3	4.5	—	77.4

**Table 9:** Minor hemoglobins in homozygous and compound heterozygous disorders (—indicates not detected).

Disorder	A1a	A1b	A1c	A2
Hb SS	1.7	—	4.6	2.7
Hb EE	1.1	0.2	3.2	90.8
Hb S *β* thal	3.4	3.8	5.5	4.2
Hb E *β* thal	3.5	6.5	5.1	54.8

**Table 10:** Minor hemoglobins in heterozygous disorders.

Disorder	A1a	A1b	A1c	A2
Hb S trait	0.9	1.03	5.4	3.3
Hb E trait	1.2	0.84	5.1	30.1
Hb D trait	0.3	0.97	4.6	3.12

**Table 11:** Thalassemias and minor hemoglobins (—indicates not detected).

Disorder	A1a	A1b	A1c	A2
*β* thal trait	1.13	1.3	5.5	5.4
*β* thal major	—	—	—	3.1
*α* thal (HbH)	7.6	0.8	3.3	1.4

**Table 12:** Comparison with other studies.

Study	Our	Sachdev et al. [8]	Rao et al. [9]	Balgir [10]
*β* thal trait	37.9	8.9	18.1	18.2
HbE trait	23.2	0.19	1.1	0.9
HbS trait	5.3	—	1.4	29.8
E *β*-thalassemia	4.6	0.23	1.3	0.7
S *β*-thalassemia	2.5	0.07	0.8	1.7
Hb SS	1.4	0.03	0.5	7.5
*β*-thalassemia major	2.3	0.6	2.9	5.3

## References

[1] Balgir RS (1996). Genetic epidemiology of the three predominant abnormal hemoglobins in India. *Journal of Association of Physicians of India*.

[2] Balgir RS (2005). The spectrum of haemoglobin variants in two scheduled tribes of Sundargarh district in north-western Orissa, India. *Annals of Human Biology*.

[3] Kamble M, Chatruvedi P (2000). Epidemiology of sickle cell disease in a rural hospital of Central India. *Indian Pediatrics*.

[4] Agarwal S, Gupta UR, Kohli N, Verma C, Agarwal SS (1989). Prevalence of haemoglobin D in Uttar Pradesh. *Indian Journal of Medical Research B*.

[5] Borgna-Pignatti C, Galanello R, Greer JP, Foerster J, Lukens JN, Rodgers GM, Paraskevas F, Glader B (2004). Thalassemias and related disorders. Quantitative disorders of hemoglobin synthesis. *Wintrobes Clinical Hematology*.

[6] Vichinsky E (2007). Hemoglobin e syndromes. *Hematology*.

[7] Bain BJ (2006). Sickle cell haemoglobin and its interactions with other variant haemoglobins and with thalassaemias. *Haemoglobinopathy Diagnosis*.

[8] Sachdev R, Dam AR, Tyagi G (2010). Detection of Hb variants and hemoglobinopathies in Indian population using HPLC: report of 2600 cases. *Indian Journal of Pathology and Microbiology*.

[9] Rao S, Kar R, Gupta SK, Chopra A, Saxena R (2010). Spectrum of haemoglobinopathies diagnosed by cation exchange-HPLC & modulating effects of nutritional deficiency anaemias from north India. *Indian Journal of Medical Research*.

[10] Balgir RS (2005). Spectrum of hemoglobinopathies in the state of Orissa, India: a ten years cohort study. *Journal of Association of Physicians of India*.

[11] Dangi CBS, Sajid M, Sawke GK, Ambhore J (2010). Sickle cell hemoglobinopathies in district Bhopal. *Indian Journal of Human Genetics*.

